# Effect of a chimney-fitted improved stove on pregnancy outcomes in Northwest Ethiopia: a randomized controlled trial

**DOI:** 10.1186/s12884-024-06363-9

**Published:** 2024-03-12

**Authors:** Habtamu Demelash Enyew, Abebe Beyene Hailu, Seid Tiku Mereta

**Affiliations:** 1 College of Health Sciences, Department of Public Health, Debre Tabor University, Debre Tabor, Ethiopia; 2 Institution of Health, Department of Environmental Health Science and Technology, Jimma University, Jimma, Ethiopia

**Keywords:** Improved stove, Intervention, Randomized control trial, Low birth weight, Preterm birth, Stillbirth, Ethiopia

## Abstract

**Background:**

Exposure to household air pollution during pregnancy has been linked to adverse pregnancy outcomes. Improved stove was implemented in Ethiopia to reduce this exposure and related health problems. However, the effects of improved stove interventions on pregnancy outcomes remains uncertain.

**Method:**

Individually randomized stove replacement trial was conducted among 422 households in six low-income rural kebeles of Northwestern Ethiopia. Pregnant women without known health conditions were recruited at ≤ 24 weeks gestation and randomized to an intervention or control group with a 1:1 ratio. A baseline survey was collected and a balance test was done. Two-sided independent samples t-test for continuous outcomes and chi-square for categorical variables were used to compare the effect of the intervention between the groups. Mean differences with 95% CIs were calculated and a *p*-value of < 0.05 was considered statistically significant.

**Result:**

In this study, the mean birth weight was 3065 g (SD = 453) among the intervention group and not statistically different from 2995 g (SD = 541) of control group. After adjusting for covariates, infants born from intervention group weighed 55 g more [95% CI: − 43 to 170) than infants born from the control group, but the difference was not statistically significant (*P* = 0.274). The respective percentages for low birth weight were 8% and 10.3% for intervention and control groups respectively (*P* = 0.346). However, the average gestational age at delivery was higher among improved stove users (38 weeks (SD = 8.2) compared to control groups 36.5 weeks (SD = 9.6) with statistically significant difference at 0.91 weeks (95% CI: 0.52 to 1.30 weeks, *p* < 0.001). The corresponding difference in risk ratio for preterm birth is 0.94 (95% CI:0.92 to 0.97; *p* < 0.001). The percentages for maternal complications, stillbirth, and miscarriage in the intervention group were not statistically different from the control group.

**Conclusions:**

While the increase in average birth weight among babies born to mothers using improved stoves was not statistically significant, babies had a longer gestational age on average, offering valuable health benefits. However, the study didn’t find a significant impact on other pregnancy outcomes like stillbirth, miscarriage, or maternal complications.

**Trial registration:**

The study was registered at the Pan African Clinical Trial Registry website under the code PACTR202111534227089, (https://pactr.samrc.ac.za/ (Identifier). The first trial registration date was (11/11/2021).

## Background

Nearly 109 million Ethiopians rely on plant or animal matters like wood, charcoal, and dung for cooking, light, and warmth [1–4]. The combustion of these solid biomass fuels through inefficient traditional stoves emits a range of air pollutants, including fine particulate matter (particles with a diameter of ≤ 2.5 μm, henceforth “PM_2.5_”) [5, 6] which is by far the most significant exposure agent impacting public health [7–10]. The size of these particles is directly linked to their potential for causing health problems where PM_2.5_ poses the greatest health risk due to its ability to get deep into the lungs and the bloodstream [11–13]. PM_2.5_ could trigger inflammation and could also reach the placenta leading to placental damage with fetal consequences [8, 11].

The extensive utilization of biomass fuels alongside with inefficient and unsafe traditional cooking stoves poses a significant public health crisis, impacting various human body systems, including the cardiovascular system [12, 13], respiratory system (acute respiratory infections, the leading cause of mortality among children under 5 years of age [14–18], pregnancy outcomes [19–24], cognitive function [25, 26] and increase eye problems [27–29]. Globally, the use of biomass fuel has resulted in over 2.3 million deaths and 91.5 million disability-adjusted life years (DALYs), with a distinct geographical variance, predominantly concentrated in Southeast Asia and sub-Saharan Africa [3, 10, 30]. The burden of diseases due to PM_2.5_ in Africa is among the highest in the world [3, 31] and Ethiopia is the second among the top 10 countries with the highest number of deaths linked to household air pollution (HAP) across Africa in 2019 next to Nigeria [10].

Beyond the cardiorespiratory impact, emerging evidences indicate a link between HAP and detrimental pregnancy outcomes [7, 32, 33] encompassing conditions such as low birth weight (LBW), pre-term birth (PTB), congenital anomalies [34, 35] and post-neonatal infant mortality [36]. Because, fetuses during pregnancy are uniquely susceptible to air pollution due to the critical stage of development they are in, where rapidly dividing cells and shifting metabolic needs make them sensitive to environmental toxins [37, 38]. Pollutants including PM_2.5_ can be absorbed into the maternal blood stream increasing risk of adverse health effects and potentially affecting fetal growth by directly crossing the placenta [11]. These outcomes are known to have implications for later health status during childhood and adulthood [7, 32, 38].

These significant adverse effects on cardiorespiratory health and birth outcomes due to the use of biomass fuels underscore the need for diverse intervention strategies [39, 40]. Among these, the distribution of improved stoves emerges as the foremost priority for effecting a safe transition to cleaner energy in impoverished rural African settings [41]. Studies suggest that interventions delivered during critical windows of fetal development, particularly during the second trimester [42], early pregnancy [43], early pregnancy and late first trimester [44] can demonstrably improve birth weight and other fetal outcomes. Furthermore, the introduction of improved stoves is anticipated to significantly contribute to advancing at least four sustainable development goals [45]; Goal 3 (good health and well-being), Goal 5 (gender equality), Goal 7 (affordable and clean energy), and Goal 13 (climate action) [45–47]. Consequently, the Ethiopian federal government has proposed to provide improved stoves to 30 million households by 2030 [48]. This has led to the operation of various types of improved stoves in different parts of the country at different times [49, 50].

Despite growing literatures linking HAP to pregnancy outcomes, only few interventions have been tested to generate evidences on effectiveness of these interventions [51–55]. Only two randomized controlled trial (RCT) studies in Ethiopia tested improved stoves for their impact on childhood respiratory illness or growth [56, 57]. Both studies implemented *Mirt* stove (known as best stove in English) without chimney deliberately designed for *Injera* preparation. Injera, a staple food for Ethiopian dish, is a flatbread similar to a pancake made from a small grain known as *Teff* (scientifically termed *Eragrostis teff*) [58]. However, the trials did not yield a significant decrease in the occurrence of acute respiratory infections [56]. But, improvement was observed in child growth [57] and in reducing the concentration of PM_2.5_ compared to the traditional stove [56].

While chimney-fitted Mirt stoves hold promise for reducing household air pollution and potentially improving health outcomes, their population-level impact on pregnancy outcomes remains inadequately evaluated. This study addresses this gap by investigating whether introducing such stoves before the third trimester of pregnancy can significantly increase newborn weight, a key indicator of infant health.

## Materials and methods

### Study settings

This study was carried out in a low-income rural community situated in the Guna -Tana integrated field research and development center of Debre Tabor University, within the South Gondar zone of Ethiopia. In this area, biomass fuel is the sole household energy source utilized for cooking, baking, and heating, typically employed with traditional three-stone stoves. Further information about the study site has been detailed in our previous publication [59].

### Study design

The study employed a parallel, household level-randomized, control trial design with a 1:1 ratio to assess the effect of chimney-fitted Mirt stove use during pregnancy on pregnancy outcomes in south Gondar zone, Ethiopia. The trial is registered with the registration date of November 11, 2021and under the registration code of (https://pactr.samrc.ac.za/: ACTR202111534227089). Households with eligible pregnant women were allocated in to two arms of equal size, either to replace their usual traditional stove with Mirt stoves (intervention), or to continue cooking with traditional stove (control). The main purpose of the study was to compare the impact of improved stove intervention with traditional stoves in terms of pregnancy outcome mainly birth weight in a live-born singleton pregnancy.

### Participants

All pregnant women who were residing in six kebeles located in the Guna-Tana integrated field research and development center were invited to participate in the study. To be eligible and participate in this study, a pregnant woman must meet the following inclusion criteria: Aged 18–38 years, in her first or second-trimester gestation (gestational age ≤ 24 weeks determined by the self-reported first day of last menstrual period (LMP) and ultrasound (as appropriate), exclusively use the traditional biomass-fueled stove, most frequently responsible for cooking in her household, carrying a live singleton fetus and previously healthy women. Household air pollution exposure in mid-and late- gestations are associated with lower birth weight risk [43, 60]. It was also evidenced that kitchen smoke exposure during the window of developmental susceptibility in early life is particularly detrimental [38, 61]. A recent findings from China indicate that the third trimester is a particularly susceptible period for PM_2.5_ exposure and its association with preterm birth [62]. But, those pregnant women who had plan to move permanently outside the study area in the next 12 months, likely to use clean stoves predominantly in the near future, engaged in local alcohol production, and do not have an enclosed main cooking area (kitchen) structure were excluded from participation in the study.

### Screening procedures

Different approaches were used to screen potentially eligible pregnant women in the study area. Regular monthly meetings with health workers provided a familiar platform to introduce the study and invite participation. These sessions are typically scheduled for the 5th day of each month at the health post, focusing on discussions related to pregnancy and childbirth with midwives. Field data collectors worked together with health extension workers and local health development leaders to visit the homes of pregnant women who may not attend the monthly meetings, ensuring comprehensive outreach. Furthermore, data from antenatal clinic (ANC) registries at healthcare facilities were utilized to identify prospective participants. Field data collectors worked with midwives from adjacent health centers, health extension workers (HEWs), and community health development army leaders (HDALs) in the whole study period.

### Sample size

All pregnant woman within the study kebeles of the Guna-Tana integrated field research and development center underwent screening to determine their eligibility for study participation. According to the actual data obtained from the district health offices and local health extension workers’ records, the anticipated count of pregnant women from six kebeles was 648. However, after excluding ineligible pregnant women based on inclusion criteria, the participant count was reduced to 422 pregnant women.

### Variables and measurements

#### Birth Weight

birth weight is the primary outcome for this study and is defined as the first weight of a newborn baby, measured within 48 h of birth (ideally 24 h) [63]. However, due to some practical challenges that made it difficult to reach the home to carry out measurements, such as delayed notification by family members and communities located distant away from the main road, we considered birth weights measured up to 48 h after birth. To ensure timely data collection, each household in both groups assigned a specific family member to contact the data collectors via mobile phone immediately upon a birth. The weight of newborn babies was collected at health centers (for institutional delivery) and at the household level (for home delivery). For institutional delivery, birth weights were measured and recorded by midwives who took sensitization training on measuring and recording at the health centers. For home deliveries, a field worker who took similar training measured the weight of the newborn babies using a regularly calibrated portable digital scale (Seca). Measuring the weight of the newborns at the household level was carried out by measuring the weight of the mother with and without carrying her baby and taking the difference as the weight of the newborn. Birth weight was measured twice to the nearest gram and the average was taken. If the two weight measurements differ by more than 10 g, then a third weight measurement was taken.

#### Low birth weight

This study also analyzes the proportion of newborns with low birth weight (LBW), defined as < 2500 g regardless of gestational age, in both intervention and control groups [64]. It is further classified as very low birth weight (less than 1500 g) and extremely low birth weight (less than 1000 g) [63].

#### Gestational age

gestational age (GA) represents the duration from the first day of the woman’s last menstrual period to the enrolment and birth dates in weeks. GA upon enrollment was determined through self-report and, in part, via ultrasound. At the time of data collection, an ultrasound screening campaign was taking place in the South Gondar zone, and with proper authorization, we were able to leverage the results.

#### Preterm birth

births were classified as preterm if the gestational age at birth was less than 37 weeks and as term if the gestational age at birth was 37 weeks or more. Stillbirth refers to fetal demise after 28 weeks of pregnancy, while miscarriage denotes fetal loss before 28 weeks of pregnancy [64].

#### Maternal complications

are conditions that include antepartum or postpartum hemorrhage, prolonged or obstructed labor, postpartum sepsis, complications of abortion, pre-eclampsia /eclampsia, ectopic pregnancy, and ruptured uterus. If one of these conditions occurs, we count it as a maternal complication.

#### Asset index

We assessed the socioeconomic status of the respondents in terms of asset index as recommended rather than using income or expenditure to characterize the the respondents. Because it is less susceptible to short-term economic shocks and likely a better proxy of longer-term household wealth [65]. Refer to our previous publication for a detailed description of the methodology used to determine the socioeconomic status of the respondents [59].

#### Dietary diversity score

Since women’s dietary intake is the proxy indicator for pregnancy outcomes, information on women’s minimum dietary diversity score (WDDs) was collected using the FAO guidelines [66]. Pregnant women were asked to recall all the solid and semisolid food items consumed in the 24 h prior to the interview, first spontaneously and then by probing to ascertain that no meal or snack was left out. Ten food groups were proposed for the women’s dietary diversity score (WDDS). The ten food groups used to calculate WDDS were starchy staples (cereals and white tubers), dark green leafy vegetables, other vitamins A rich fruits and vegetables, other fruits and vegetables, organ meat, meat and fish, eggs, legumes, nuts and seeds, milk and milk products. Pregnant women were then categorized as consuming either adequate dietary diversity (≥ 5 food groups) or inadequate dietary diversity (< 5 food groups).

#### Maternal body mass index

maternal height and weight were measured upon enrollment, providing the data required to calculate the maternal body mass index (BMI), which is derived by dividing the weight in kilograms by the square of the height in meters (kg/m2).

#### Improved water source

improved water sources are defined as those that are likely to be protected from outside contamination, and fecal matter in particular [67]. In this study, protected dug wells, protected springs, and community standpipes were considered as improved water sources.

#### Improved sanitation facilities

an improved sanitation facility is one that likely hygienically separates human excreta from human contact [67]. In this study, pit latrines with slabs and/or ventilation were considered as improved sanitation facility.

### Intervention

The intervention in this trial was replacing the traditional stoves that are used earlier in the enrolled pregnant women’s household in the intervention group with a locally sourced and chimney-fitted improved stove (locally named as Mirt stove, which means best stove). With their husbands’ consent, participants in the intervention group chose suitable spots for their new stoves. Trained local producers built and installed the Mirt stoves, moving from house to house in each Kebele (Ethiopian smallest administrative unit) within two weeks of randomization. These skilled installers also provided repair services throughout the trial.

**Fig. 1:**
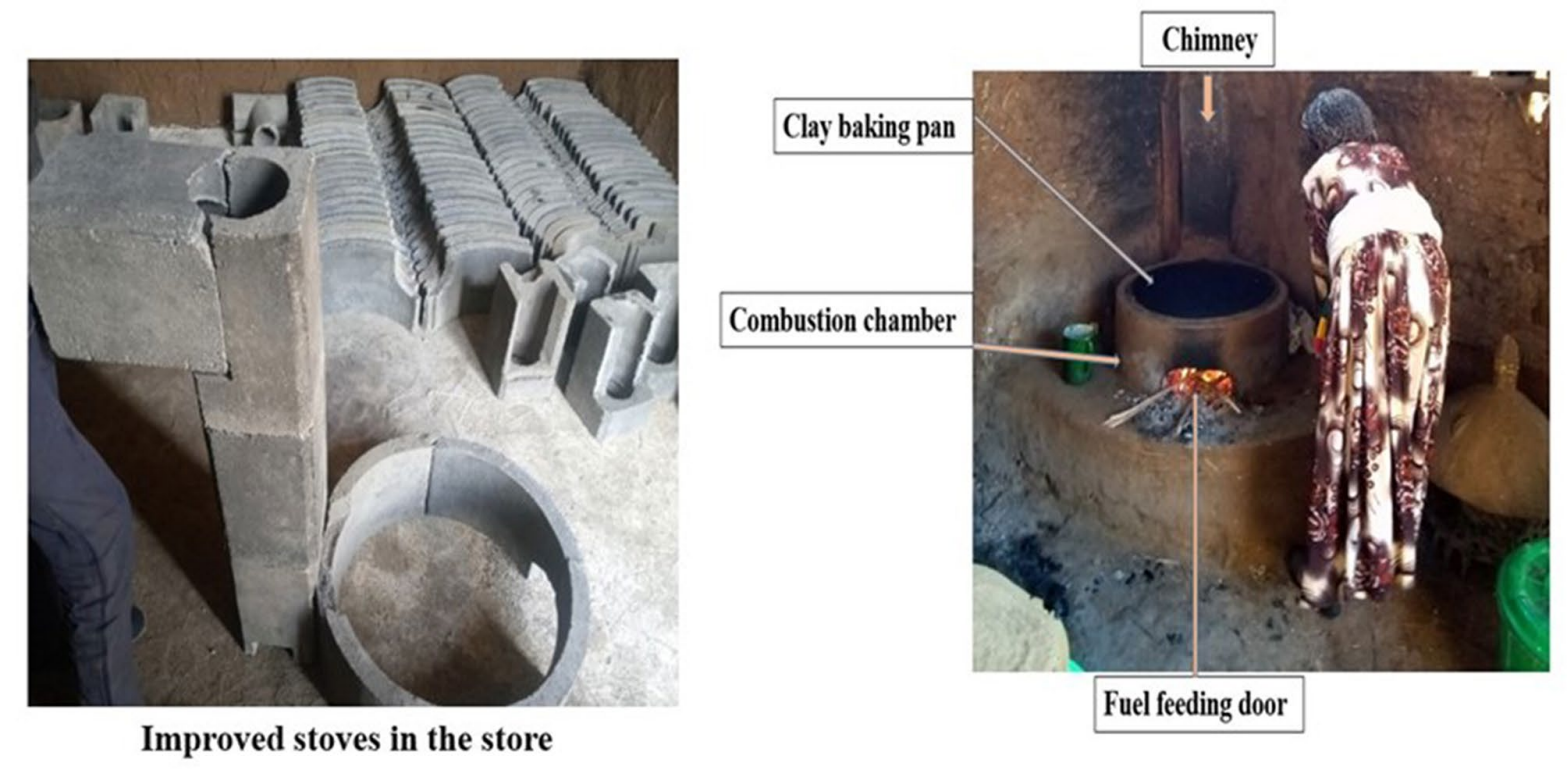
Chimney fitted Mirt stove technology in the stock before installation and after the installation

The key features of the stove are a clay baking pan, an enclosed combustion chamber to traps heat and improves fuel efficiency and reducing wood consumption, and a chimney to vents smoke and harmful emissions out of the kitchen, improving indoor air quality and health (Fig. 1). The chimney was designed attached to the back of the combustion chamber to vent the smoke through the wall to the outdoors. The baking cooking surface is a clay plate used for baking Injera. The front of the stove has a door for fuel feeding. The stoves were constructed by trained local producers who built and installed them by moving from house to house.

The Mirt stove is well-accepted, culturally appropriate, locally sourced, and functions well when maintained correctly. It has a specific fuel consumption reduction of 40 to 50% compared to the open-fire traditional stove [49]. Previously, a similar stove without a chimney was reported to reduce indoor PM_2.5_ by 58% [56] and exhibited an enhancement in child growth [57]. However, earlier trials demonstrated a non-significant decrease in the incidence of acute respiratory infections compared to the continued use of a traditional open-burning baking stove method [56].

The stoves were procured from a local producer for the unit market price of Ethiopian birr 400 (approximately US dollars (USD) $8) and provided with half subsidized to study participants. All intervention stoves were deployed during December 2022. Health extension workers and local health development army leaders diffused behavior-based messaging to pregnant women for the exclusive use of the improved stove and checked stove condition during their regular home visits. The whole trial was ended when new born babies reached to 6 months of age due to time and logistics restriction.

### Randomization

Following the acquisition of informed consent and the completion of baseline assessments, study participants were randomly assigned in a 1:1 ratio to either use a chimney-fitted Mirt stove or to continue using a traditional three-stone stove (control group), employing an Excel random number generator. This process was primarily designed to ensure that any potential confounding factors, whether known or unknown, were evenly distributed across each of the intervention groups, thus preventing bias in the comparison of outcome measures between the groups. The allocation sequence was generated by the invited biostatistician, who also determined the enrollment of participants and their assignment to interventions. As blinding was not feasible in certain circumstances, the nature of this trial made blinding regarding the type of intervention unfeasible.

### Data collection

A structured paper format questionnaire was administered through face-to-face interviews by trained field data collectors with the assistance of health extension workers and local health development army leaders in each Kebele. The baseline survey covered a range of topics including socio demographic and economic status, housing characteristics, kitchen types, cooking behaviors, fuel type, sources of drinking water, household sanitation practices, and pregnancy-related information. Pregnant women were also surveyed about their health status, including medication use and all of the other characteristics that are potentially associated with pregnancy outcomes. Information on women’s dietary diversity score (WDDs) was collected using FAO women’s dietary diversity score guidelines [68]. After the detailed baseline survey was completed and all eligible pregnant women were assigned to the intervention or control arm, pregnancies were tracked to their birth outcomes. Concurrently, follow-up data were collected on stove use and maternal health conditions for both intervention and control groups. Stove use was monitored throughout the study period in both intervention and control households using a combination of observations and interview reports.

The assigned family members were asked to notify as soon as possible to the local health development leaders, data collectors, or health extension workers if the enrolled woman went into labor. After birth, we obtained birth weight, gestational age, sex of the newborn, and mode of delivery. Furthermore, the family was requested to inform any form of pregnancy outcome including miscarriage.

### Data quality control

Comprehensive data quality control measures were implemented to ensure the accuracy and reliability of the collected information. Data collectors received training on calibrating and using standardized Seca scales to minimize instrument-related errors. A pre-test conducted at both health facilities and community levels further mitigated inter- and intra-observer inconsistencies. Interviewers underwent training to minimize misinterpretation and maintain consistent methodology including a detailed discussion to ensure consistent application of FAO guidelines for collecting 24-hour dietary intake data, promoting reliable assessment across participants. Supervisors conducted random 5% duplicate home visits to independently verify information collected by fieldworkers, identifying any discrepancies. Regular feedback sessions addressed challenges and inconsistencies encountered during data collection, refining the process and maintaining data quality.

### Data analysis

Descriptive statistics on socio-geographic and economic characteristics as well as cooking activities are calculated and presented. Baseline data were summarized by frequencies and percentages for categorical variables and by means and standard deviations for continuous variables. These characteristics were compared between households that were randomized to the intervention and those randomized to the control to assess comparability between the two groups.

The mean birth weight was compared between the intervention and control arms using independent sample t-tests and chi-square tests for low birth weight and other categorical variables. Mean differences with 95% confidence intervals (CIs) were calculated. A *p*-value of < 0.05 was considered statistically significant for hypothesis testing. However, adjustment was done for covariates with an observed statistical association (*p* < 0.25) with either outcome or exposure to control the effect of these variables on the outcome variable (mean birth weight).

In addition to overall comparisons of mean birth weight, subgroup comparison of differences in mean birth weight between the two arms by infant sex, gestational age, kitchen roof type, time spent in the kitchen per day, and pregnancy period at which intervention was introduced. We additionally conducted secondary analyses for primary outcomes (low birth weight) and secondary outcomes including preterm birth, stillbirth, and miscarriage and compared using risk ratios and 95% confidence intervals from poison regression models with a log link and robust variance. In addition to unadjusted comparisons, an adjusted comparison was made after adjusting for maternal age, dietary diversity score, maternal and paternal education, parity, and maternal body mass index. Adjustment was made on these variables on which the group difference at baseline was significant at *p* < 0.25. The mean birth weight was also estimated for subgroups stratified by newborn sex, kitchen roof type, time spent in the kitchen per day, gestational age at birth (grouped as preterm and term birth), and gestational age at which intervention was introduced.

## Result

### Participant eligibility and randomization

**Fig. 2:**
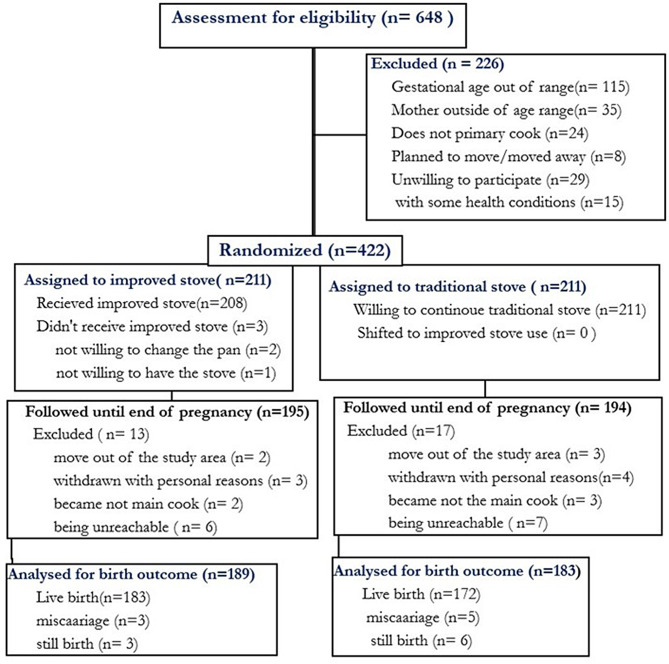
The Consolidated Standards of Reporting Trials (CONSORT) flow diagram

Between November and December 2022, a total of 648 pregnant women were assessed for eligibility in the six kebeles of the Guna-Tana integrated field research and development center, South Gondar zone. Out of these, 226 pregnant women were excluded due to reasons such as unwillingness to participate (*N* = 29), being outside the age range (*N* = 35), having certain health conditions (*n* = 15), planning to permanently move to a nearby town (*n* = 8), not primarily being responsible for cooking (*n* = 24), and having a detected gestational age exceeding 24 weeks (*N* = 115) (refer to Fig. 2). Finally, 422 households with eligible pregnant women were enlisted for the study and underwent randomization, with 211 assigned to the intervention group and 211 assigned to the control group. However, 3 participants from the intervention group did not receive the improved stove, and no case was observed of control households switching to improved stoves during the study period. Throughout the study, 30 pregnant women (13 from the intervention group and 17 from the control group) were excluded due to lost to follow-up, non-adherence, and withdrawn.

### Maternal and infant characteristics

The average age of participants at enrollment was 28.9 ± 5.3 years with the largest group (47%, *n* = 198) falling between 25 and 31 years old. There was no significant difference in average age between the control and intervention groups. Around two-third (62%, *n* = 265) of participants were unable to read and write and nearly all participants were married at the time of enrollment. None of the participating woman was smoker or living with a smoker at enrollment. Participants were enrolled at an average of 18 weeks pregnant (range: 12–24 weeks) and there was no significant difference in average gestational age between the control and intervention groups. Over half (54%, *n* = 231) of participants enrolled in their second trimester with no significant difference between the control and intervention groups. The average number of previous live births was 2.7 + 1.6 (2.6 vs. 2.7) for intervention and control groups respectively (*p* = 0.613).

The average dietary diversity score (DDs) within the last 24-hours data collection period was 3.87 and the specific values for control and intervention participants were 3.94 and 3.81, respectively. More than three-fourths (76%, *n* = 321) of the participating pregnant women (74.9%, *n* = 158, in the control group and (77.3%, *n* = 163, in the intervention group) consumed inadequate dietary diversity. Both the intervention and control groups received an average of two ANC visits, and 17.1% of the intervention group and 15.6% of the control group had experienced a prior miscarriage.

Covariate imbalance was checked using χ2 tests for categorical variables and independent sample t-test for continuous variables mainly to check the success of randomization. As shown in Table 1, the study groups demonstrated balance in the key baseline characteristics such as maternal age at enrollment, marital status, maternal and paternal educational status, family size, asset index, gestational age at enrollment, dietary diversity score, body mass index and other characteristics indicating our randomization achieved reasonable balance other than intervention stove.

**Table 1:** Baseline demographic and cooking characteristics by intervention arms among pregnant women in south Gondar zone, northwestern Ethiopia

Characteristics	Control (*n* = 211)		Intervention (*n* = 211)		*P*-Value*
Maternal age at entry, yrs (Mean ± SD)	29.2 ± 5.3	-	28.6 ± 5.1	-	0.244
18–24	35	16.7	44	21.0	0.451
25–31	99	47.1	99	47.1	
32–38	76	36.2	67	31.9	
Marital status, n( %)					
currently married	201	95.3	204	96.7	0.458
currently not married	10	4.7	7	3.3	
Maternal education level, n( %)					
unable to read and write	142	67.3	123	58.3	0.073
read and write only	42	19.9	62	29.4	
primary and above grade	27	12.8	26	12.3	
Paternal education level, n ( %)					
unable to read and write	92	43.6	72	34.1	0.080
read and write only	74	35.1	95	45.0	
primary and above grade	45	21.3	44	20.9	
Family size, Mean, SD	4.7 + 1.6	-	4.7 + 1.5	-	0.952
< 5 occupants	95	45.0	96	45.5	0.500
>= 5 occupants	116	55.0	115	54.5	
Had under-five children					
yes	85	40.3	91	43.1	0.622
no	126	59.7	120	56.9	
GA at entry (weeks), Mean ± SD	17.8 ± 3.8	-	18.1 ± 3.8	-	0.501
Birth order					
primigravida	22	10.4	12	5.7	0.074
multigravida	198	89.6	199	94.3	
Prior miscarriage					
yes	36	17.1	33	15.6	0.793
no	175	82.9	178	84.4	
Dietary diversity scores, mean (SD)	3.9 + 0.9	-	3.8 + 0.9	-	0.154
adequate (≥ 5 food groups)	53	25.1	48	22.7	0.324
inadequate (< 5 food group)	158	74.9	163	77.3	
Currently, alcohol drinking					
yes	112	53.1	99	46.9	0.461
no	119	56.4	91	43.1	
BMI (kg/m2), mean (SD)	20.9 ± 2.3	-	20.8 ± 1.9	-	0.518
Asset index, n(%)					
low	68	32.2	71	34.1	0.387
medium	77	36.5	63	30.3	
high	66	31.3	74	35.6	
Cooking time spent, hrs, mean (SD)	2.7 ± 0.9	-	2.8 ± 0.9	-	0.558
Number of ANC visits mean (SD)	1.9 ± 0.9		1.9 ± 0.7		0.952
Drinking water sources, n(%)					
improved	57	27.0	62	29.4	0.333
unimproved	154	73.0	149	70.6	
Sanitation facility, n(%)					
improved	48	22.7	57	27.0	0.284
unimproved	163	77.3	154	73.0	
Handwashing facility, n(%)					
yes	36	17.1	34	16.1	0.448
no	175	82.9	177	83.9	

*Intervention and control groups were compared using χ2 - tests for categorical variables and independent sample t-tests for continuous variables

.

### Estimating the impact of the intervention

Of the 422 women randomly assigned to groups, 398 completed the trial and provided pregnancy outcomes. However valid birth weights were measured for 359 newborns. In the Mirt stove group, 183 babies (92.9%) had their weight measured, while in the control group, it was 172 babies (90.2%). Home deliveries were slightly more common in the control group (11.4%, *n* = 19) compared to the intervention group (8.2%, *n* = 16). The gender distribution was similar, with (50%, *n* = 95) boys in the intervention group and (53%, *n* = 98) in the control group.

As depicted in Table 2, the average birth weight of babies born to mothers using the Mirt stove (3065 g (SD = 453 g) was slightly higher compared to those born to mothers using open fires (2995 g (SD= 541 g). However, this difference of 69 g (95% CI: − 31, 170; *P* = 0.153) was not found to be statistically significant. This remained true even after accounting for factors such as age, diet, education, previous pregnancies, and maternal BMI, with the adjusted difference reduced to 55 g (95% CI: -43, 153; *p* = 0.274), and still not statistically significant.

Although fewer babies born to mothers using improved Mirt stoves had low birth weight (8.1%) compared to those born to mothers using open fires (10.3%), this difference was not statistically significant. The estimated rate of low birth weight was only 2% lower among Mirt stove users (adjusted RR = 0.98; 95% CI: 0.96, 1.02; *p* = 0.419), and the difference wasn’t large enough to consider it conclusive. Based on this study, using a chimney-fitted improved stove during pregnancy may not significantly reduce the risk of low birth weight in newborns.

We also analyzed secondary endpoints, including gestational age, miscarriage, stillbirth, and maternal complication. The average AG at delivery by study arm was: 38 weeks for improved stove arm and 37 weeks for controls. The unadjusted and adjusted difference in average gestational age at delivery were 0.93 weeks (95% CI: 0.48,1.39; *p* < 0.001) and 0.91weeks (95% CI: 0.52 to 1.30; *p* < 0.001) as compared with control newborns, respectively. In addition, unadjusted analyses suggested there were fewer preterm birth infants (< 37 weeks) in the improved biomass cook stove arm compared with control (5 (2.6%) vs. 21 (11.3%) preterm births, or a risk ratio of 0.95 (95% CI: 0.93 to 0.98, *p* < 0.001); the adjusted difference was 0.94 (95% CI: 0.92, 0.97; *p* < 0.001). There were 3 (1.4%) and 5 (2.4%) miscarriages (fetal losses at < 28 weeks gestational age) and 3 (1.5%) and 6 (3.1%) stillbirths (lost or not born alive ≥ 28 weeks gestational age) in the intervention and control arms respectively. But the numbers were small and neither miscarriage nor stillbirth was statistically significant. No statistically significant difference was observed between the intervention and control groups concerning maternal complications.

**Table 2:** Associations between intervention arms and pregnancy outcomes among pregnant women in south Gondar zone, northwest Ethiopia

Pregnancy outcomes	Observed outcomes		Unadjusted difference*		Adjusted difference**	
	Intervention	Control	Difference (95% CI)	*p*-value	Difference (95% CI)	*p*-value
Mean birth weight in grams, SD	3065 ± 453	2995 ± 541	69 [ -31, 170 ]	0.175	55 [-43, 153 ]	0.274
range	1700–4200	1315–4162				
missing	13	17				
Gestational age at birth, weeks	38.0 ± 8.2	36.5 ± 9.6	0.93 [ 0.48 ,1.39 ]	*p* < 0.001	0.91 [ 0.52, 1.30 ]	*p* < 0.001
range	34–42	32–42				
missed	9	13				
Low birth weight, n(%)					.	
< 2500 g	16/198 [8.1]	20/194 [10.3]	0.988 [0.959, 1.018]	0.445	0.985[0.956, 1.016]	0.346
> 2500 g	182/198 [91.9]	174/194 [89.7]				
missed	13	17				
Preterm birth, n(%)						
< 37 weeks	5/189 [2.6]	21/186 [11.3]	0.956 [0.931, 0.982]	0.001	0.947 [0.922, 0.974]	*p* < 0.001
≥ 37 weeks	184/189 [97.4]	165/186 [88.7]			.	
missing	22	25				
Miscarriage, n (%)						
yes	3/202[1.4]	5/204[2.4]	1.650[0.400, 6.814]	0.489	1.534[0.381, 6.177]	0.548
no	199/204[94.3	199/204[94.3				
missed	9	7				
Still birth, n(%)						
yes	3/196 [1.5]	6/192 [3.1]	0.992 [0.977, 1.007]	0.299	0.988 [0.970, 1.005]	0.165
no	193/196 [98.4]	186/192 [96.8]				
missed	15	19				
Maternal complications, n(%)						
yes	31/182 [17.0]	26/194[13.4]	1.020[0.980, 1.061]	0.328	1.015[0.976, 1.056]	0.458
no	151/182 [83.0]	168/194[86.6]				
missed	29	17				

*Differences for continuous variables (birth weight and gestational age) come from linear regressions. Estimates shown represent differences in the mean of the study group and 95% CI as compared with the control group. Differences for categorical variables (low birth weight, pre-term birth, stillbirth, miscarriage, and maternal complications) come from Poisson regressions with robust estimates. Estimates shown represent risk ratios and 95% CIs as compared with the control group. Differences replicate unadjusted models and additionally adjust for maternal age, dietary diversity, maternal and paternal education, maternal body mass index, and gravidity

**Fig. 3:**
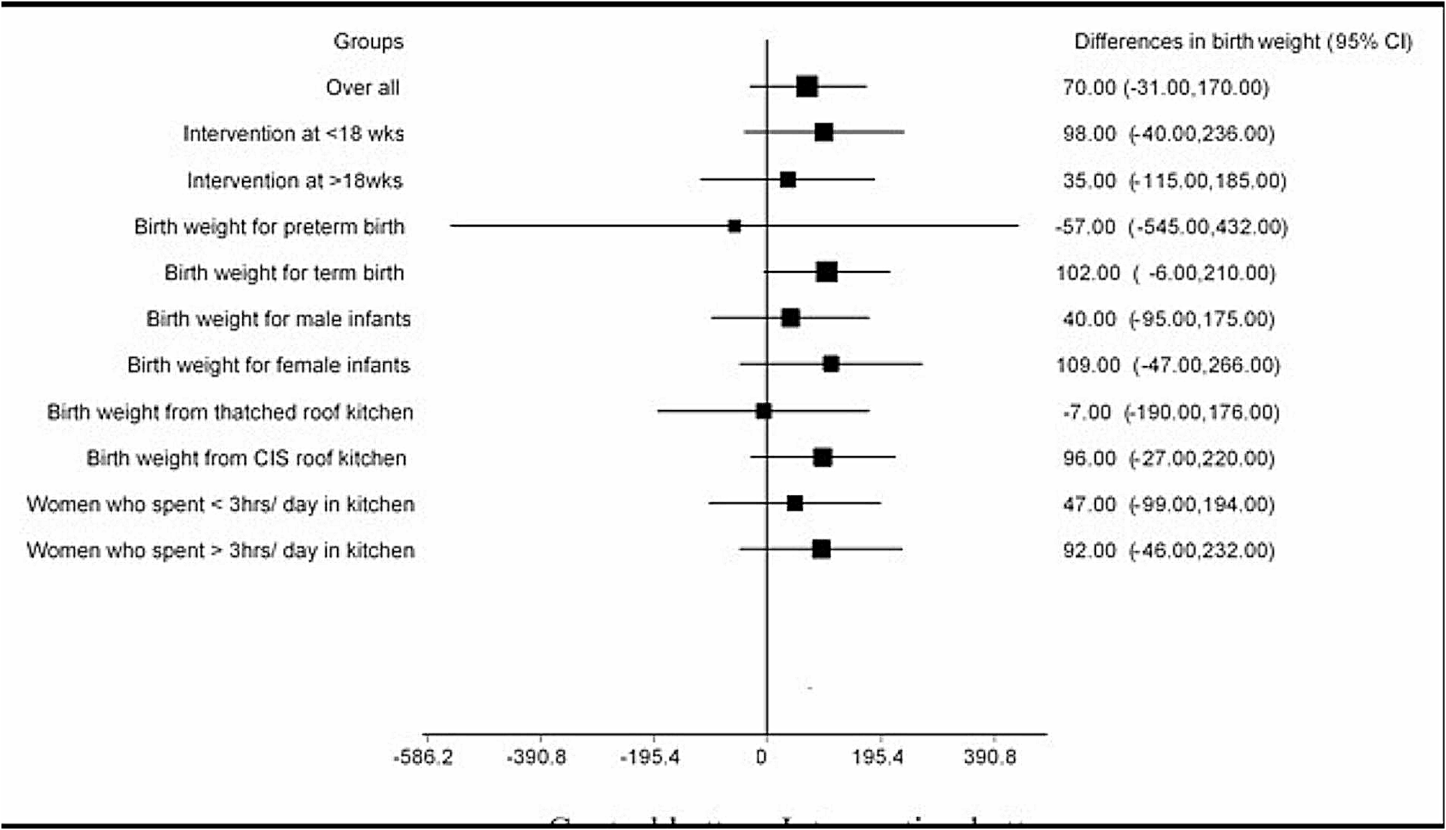
A forest plot showing subgroup analyses of the difference in birth weight between the intervention and control groups

Looking deeper into the data, we examined birth weight in various sub-groups including baby’s sex, kitchen roof type, daily time spent in the kitchen, gestational age at birth, and timing of intervention introduction. Figure 3 shows that while female babies born to mothers using Mirt stoves weighed an average of 109 g (95% CI: − 47 to 266; *P* = 0.167) more (though not statistically significant), the difference for male babies was only 40 g (95% CI: -95 to 175; *p* = 0.562). Similarly, earlier intervention (before 18 weeks) was associated with a non-significant 98 g (95% CI: -40 to 236; *p* = 0.158) increase in birth weight. Kitchen roof type didn’t significantly impact the Mirt stove’s effect, but a noticeable trend emerged for corrugated iron roofs. Babies born to mothers using Mirt stoves in these kitchens were an average of 96 g (95% CI: -27 to 220; *P* = 0.120) heavier than those in the control group. The same held true for mothers spending over three hours daily in the kitchen, with a non-significant 92 g (95% CI: -46 to 232; *p* = 0.191) increase in average birth weight.

## Discussion

In this randomized control trial (RCT), a chimney-fitted Mirt stove intervention was initiated during the first and second trimesters of pregnancy in typical rural households of northwest Ethiopia. Since birth weight is a good summary measure of multifaceted public health problems influencing the course of health outcomes over the lifespan [69–71] and considering its association with smoke from biomass fuel usage [43, 72, 73] was chosen as the primary outcome for this study. In addition, secondary analysis of the primary outcome (low birth weight) and secondary endpoints including stillbirth, miscarriage, gestational age, and maternal complications were analyzed.

Though birth weight was slightly higher in the intervention group, the difference was not statistically significant. We found an effect size of 69 g for mean birth weight and only a 2% reduction in LBW in the intervention arm compared to the control arm. After accounting for potential influencing factors, the association between the intervention and birth weight further diminished to 55 g. Despite evidences linking household air pollution to reduced birth weight [19, 64, 74–76], our study, which implemented a specific mitigation intervention, did not find a conclusive link between improved stove use and birth weight. Even evidences from systematic reviews and meta-analyses showed that exposure to household air pollution increases the risk of low birth weight [7, 77, 78].

.

Different mixed results have been reported from previous studies conducted on the impact of improved cook stoves on birth weight [53, 79, 80]. Similar inconclusive result was reported from Guatemala, which demonstrated that unadjusted and adjusted birth weight was 68 and 89 g greater among the intervention group compared with control group [79]. In Mongolia, the introduction of portable high-efficiency particulate air (HEPA) filters during pregnancy showed no association with birth weight, resulting non-significant 18 g increment [81]. While mothers who used LPG stoves in a separate large-scale study across four countries had babies with an average birth weight 19.6 g higher than those born to mothers using open fires, the difference wasn’t statistically significant [82]. Evidences from Nepal and Ghana indicated that introducing LPG or enhanced biomass cook stoves during pregnancy did not result in improved birth weight [53, 83]. The sole statistically significant birth weight increase came from an ethanol stove study in Nigeria, with an average gain of 128 g after accounting for other factors [54].

Our sub-group analyses suggest potential benefits of Mirt stoves for specific groups, even though these findings were not statistically significant. Similarly, a Mongolian study revealed that the use of HEPA filter air cleaners was associated with an 85-gram increase in mean birth weight among babies born at term in a subgroup analysis [81]. Likewise, the difference in birth weight between the groups seemed to be slightly high among infants born to women who received the intervention before 18 weeks of gestation [82]. A meta-analysis of studies examining improved stove interventions during the third trimester of pregnancy found a significant increase in average birth weight [84]. Therefore, while cleaner stoves alone might not fully protect health, studying who benefits most can improve interventions [51, 85].

Our secondary analysis revealed only a 2% decrease in low birth weight infants among women using improved stoves compared to open fire users, but this difference did not reach statistical significance. The result aligns with findings from comparable studies carried out in Nepal [53], Ghana [83], and Guatemala [79], where the introduction of an improved stove during pregnancy did not demonstrate a statistically significant reduction in low birth weight compared to an open-burning stove. While another large-scale interventional study reported no effect on low birth weight with improved stoves [52], other works from Nigeria [54], Malawi [86], and pooled estimates from systematic review and meta-analysis [87, 88] suggest the improved stoves may help in reducing low birth weight. Furthermore, a very recent studies presented an evidence of the positive impact of the stove intervention on low birth weight [86, 89].

In this study, the lack of intervention impact also extended to secondary endpoints like stillbirth, miscarriage, and maternal complications. This aligns with findings from systematic reviews conducted in LMICs, which reported inconclusive or null results for preterm birth and stillbirth reduction with improved stoves [87, 90, 91]. A large scale multi-country study, using improved stoves during pregnancy also showed no difference in miscarriage, high blood pressure problems, or severe bleeding after birth compared to open fires [52]. Similarly, no statistically significant differences were observed for post-partum hemorrhage, pre-term birth, low birthweight, hypertensive disorders of pregnancy, or small for gestational age in the Ghanaian study [33]. Neither the Guatemala study [79] nor the Ghanaian cohort study [33] found significant impacts on miscarriage rates. Though, the number of cases was small, and the difference was not statistically significant, an ethanol cook stove study in Nigeria showed a decrease in stillbirths and miscarriages among women who used the improved stoves compared to the control group [54]. But, in contradict to our result, risk reductions for stillbirth was reported from pooled estimates of previous systematic review and meta-analysis [88].

Our study uncovered a significant difference in gestational age between babies born to mothers using improved stoves during pregnancy and those born to mothers using traditional open fires. Consequently, babies in the improved stove group were delivered, on average, nearly a full week later (38 vs. 37 weeks) which is supported by strong statistical evidence (*p* < 0.001). Our result aligns with Nigerian study where women using ethanol-burning stoves had babies born with higher average gestational ages compared to those using traditional cook stoves (*p* = 0.015). However, their study observed a non-significant decrease in preterm births (*p* = 0.22) [54]. A recent review report found that using gas stoves or heaters, compared to polluting fuels like wood or coal, was significantly lowered the risk of preterm birth (p= ·033) [92]. Strategies used to protect pregnant women from cigarette smoke exposure were also proposed as potential interventions to reduce household air pollution, suggesting possible shared approaches [93]. A study in U.S. reported a notable decrease in preterm birth rates during the COVID-19 pandemic compared to the pre-pandemic period [94]. The researchers suggest several potential explanations, including reduced work hours, and lessened exposure to air pollution.

In contrary, the large scale multi-country household air pollution intervention network trials found that switching to liquefied petroleum gas (LPG) stoves and fuel did not significantly reduce the risk of preterm birth (PTB) or increase the duration of pregnancy in participating women [52, 95]. Another interventional study, focusing on improved biomass stoves in Nepal, did not demonstrate any evidence of reducing adverse birth outcomes, including preterm births [96]. It was also reported that use of various improved stove or energy did not show any association with a decreased incidence of preterm birth or small for gestational age in Mongolia [81], in Nepal [53], and in Ghana [83]. The additional demands of Injera baking on traditional stoves in our study, including increased fuel needs, longer cooking times, and higher workload, could independently contribute to stress and potentially increase the risk of preterm birth in pregnant women. This hypothesis is supported by existing research, which has identified workload and stress as independent risk factors for preterm birth [97–99]. In addition to pollutant reduction, improved stoves offered multiple benefits, including reducing biomass fuel consumption, saving time, and consequently, alleviating workload for users, as reported in other studies [100–102].

As evidenced by our subsample data, the Mirt stove offered some biomass smoke reduction as compared to open fires, but it failed to consistently lower PM_2.5_ levels in kitchens below WHO safety thresholds [103]. This limited smoke reduction may explain the absence of statistically significant improvements on pregnancy outcomes as observed in our study. This aligns with findings from most prior research, where insufficient biomass smoke reduction is often cited as a reason for inconclusive results on stove interventions and pregnancy outcomes [51, 104–108]. Clearly, the success or failure, but mostly failure of improved stoves extends beyond technology alone. Additional factors like adoption rates, cooking habits, lifestyle choices, consistent stove use, presence of other pollution sources, and the stove’s suitability for local needs all play crucial roles [109–112]. In our study, the Mirt stove’s design, optimized for the energy-intensive practice of Injera baking (done 2–3 times weekly in Ethiopia), may have limited its impact on overall household air pollution, as other cooking activities likely involved different stoves and additional pollutant emissions [113]. Furthermore, the multifactorial nature of adverse pregnancy outcomes, influenced by factors like genetics, nutrition, and unknown etiologies, may further contribute to the null findings in our study [114–116].

### Limitations

While this RCT addressed crucial research gaps and implementing a standardized protocol for assessing improved stove impacts on pregnancy outcomes, it also faced certain limitations. First, relying on last menstrual period for gestational age estimations could have introduced some misclassification of preterm births. Second, the sample size, determined by the available eligible pregnant women in our research area, might not have been sufficient to detect less frequent events like the specific adverse pregnancy outcomes assessed. Third, stove distribution at an average of 18 weeks gestation (second trimester) may have limited the intervention’s potential influence on first-trimester exposure. Fourth, though seasonal variations in food insecurity and exposure levels might influence pregnancy outcomes, we focused on overall effects and did not analyze seasonal differences. Fifth, despite offering benefits, our intervention stove’s limited use for certain cooking activities compels women to rely on additional stoves, potentially exposing them to harmful pollutants from these alternative sources. Finally, both participants and field data collectors were aware of intervention assignment (not blinded), raising the possibility of bias in self-reported adherence and health outcomes.

## Conclusion and recommendations

While our study did not find statistically significant effects on birth weight, it revealed encouraging associations with increased gestational age and a notable reduction in preterm birth rates among women using Mirt stoves. These findings, representing potentially significant reductions in infant mortality and long-term health risks, highlight the limitations of stoves optimized solely for Injera preparation. Future iterations should prioritize multi-functionality to address diverse cooking practices (coffee, sauce) and fuel sources. Additionally, further research with larger sample sizes and longer durations is needed to explore the mechanisms behind these observations and evaluate the effectiveness of comprehensive interventions targeting all household air pollution sources. Recognizing the multifaceted nature of the problem, prioritizing interventions that address the broader cooking landscape, through research collaborations and diverse funding opportunities, holds significant potential for improving maternal and child health outcomes in communities exposed to household air pollution.

## Data Availability

The datasets used and/or analyzed during the current study are available from the corresponding author on reasonable request.
