# Correction: Effect of a chimney-fitted improved stove on pregnancy outcomes in Northwest Ethiopia: a randomized controlled trial

**DOI:** 10.1186/s12884-024-06676-9

**Published:** 2024-07-17

**Authors:** Habtamu Demelash Enyew, Abebe Beyene Hailu, Seid Tiku Mereta

**Affiliations:** 1 College of Health Sciences, Department of Public Health, Debre Tabor University, Debre Tabor, Ethiopia; 2 Institution of Health, Department of Environmental Health Science and Technology, Jimma University, Jimma, Ethiopia

**Correction: BMC Pregnancy Childbirth 24, 192 (2024)**

10.1186/s12884-024-06363-9

Following publication of the original article [1], the authors reported an error in the affiliations of author Habtamu Demelash Enyew; and in figs. 1 and 2.

Below is the correct Fig. 1.

**Fig. 1:**
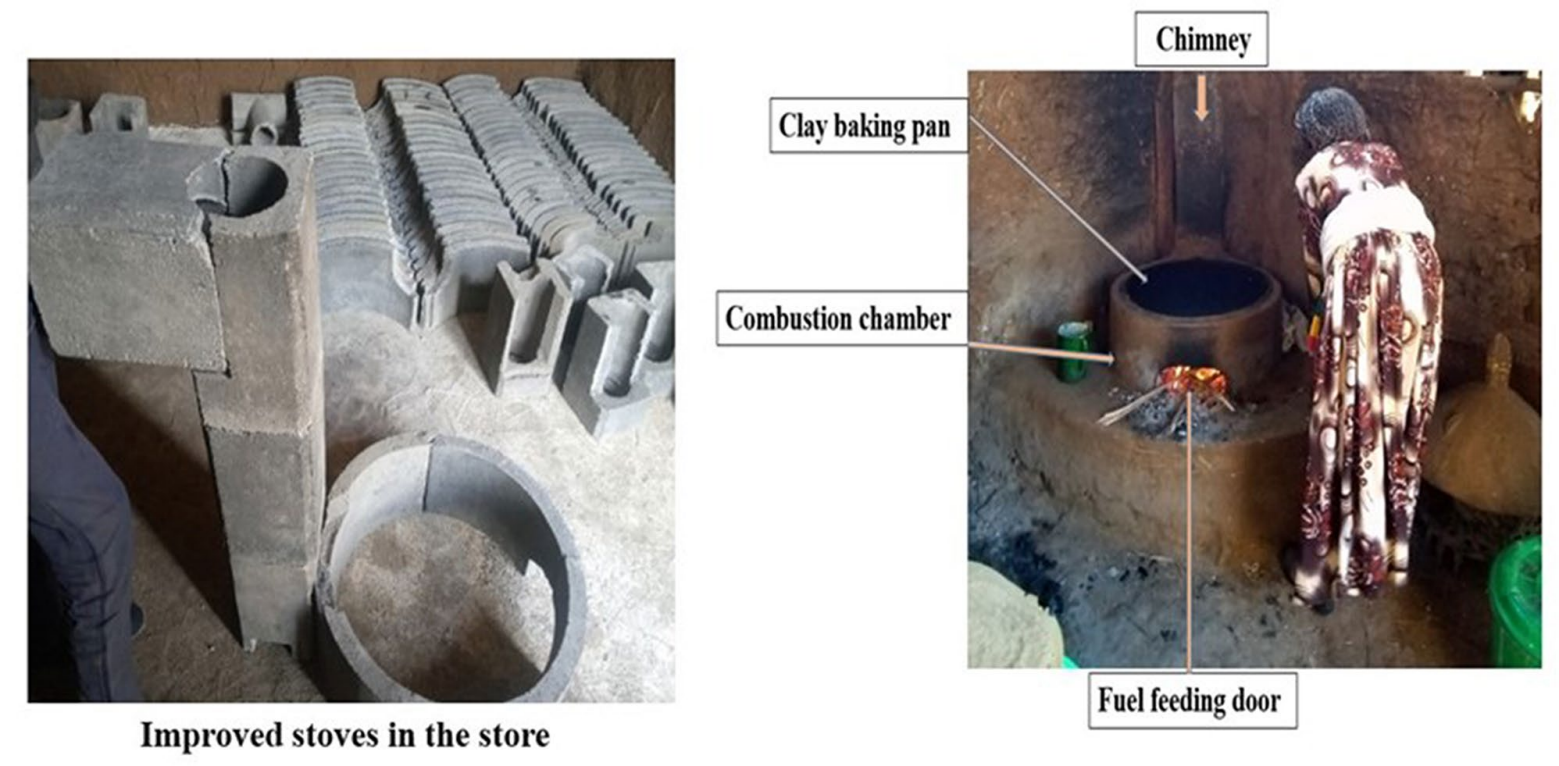
Chimney fitted Mirt stove technology in the stock before installation and after the installation

Below is the incorrect Fig. 1.

**Fig. 1:**
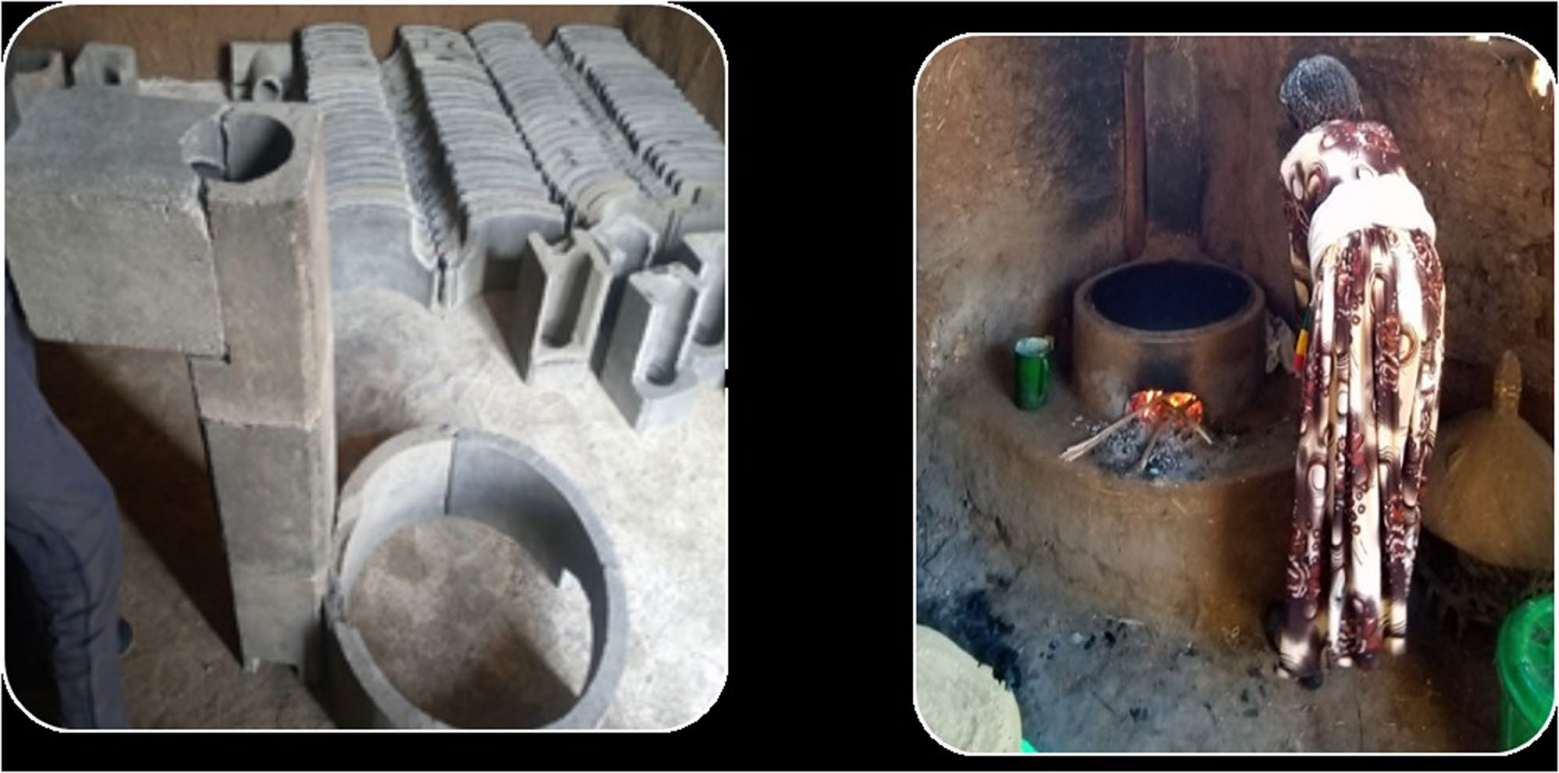
Chimney fitted Mirt stove technology in the stock before installation and after the installation

Habtamu Demelash Enyew^1,2^ should have been listed instead of Habtamu Demelash^1^.

Below is the correct Fig. 2

**Fig. 2:**
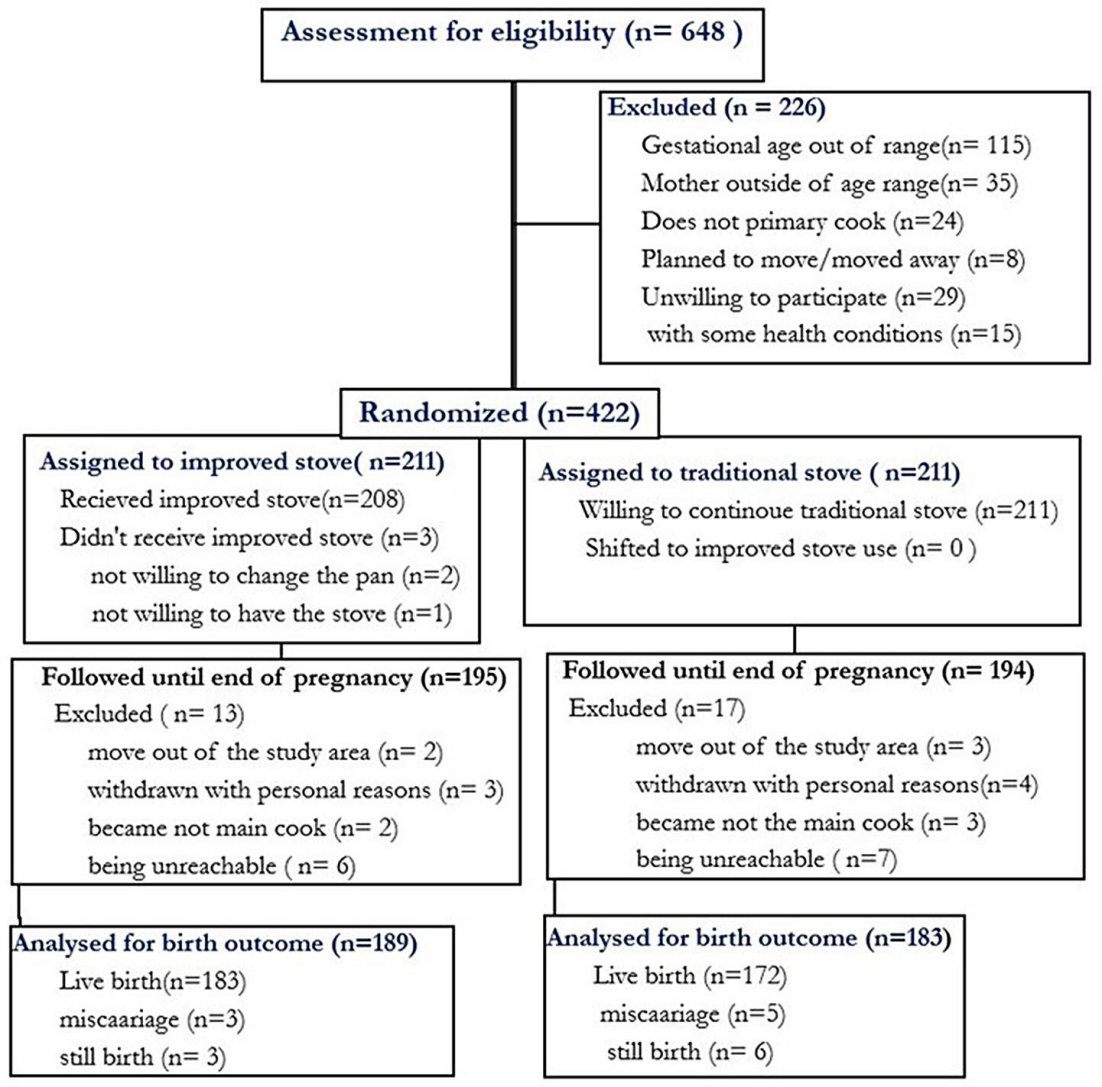
The Consolidated Standards of Reporting Trials (CONSORT) flow diagram

Below is the incorrect Fig. 2.

**Fig. 2:**
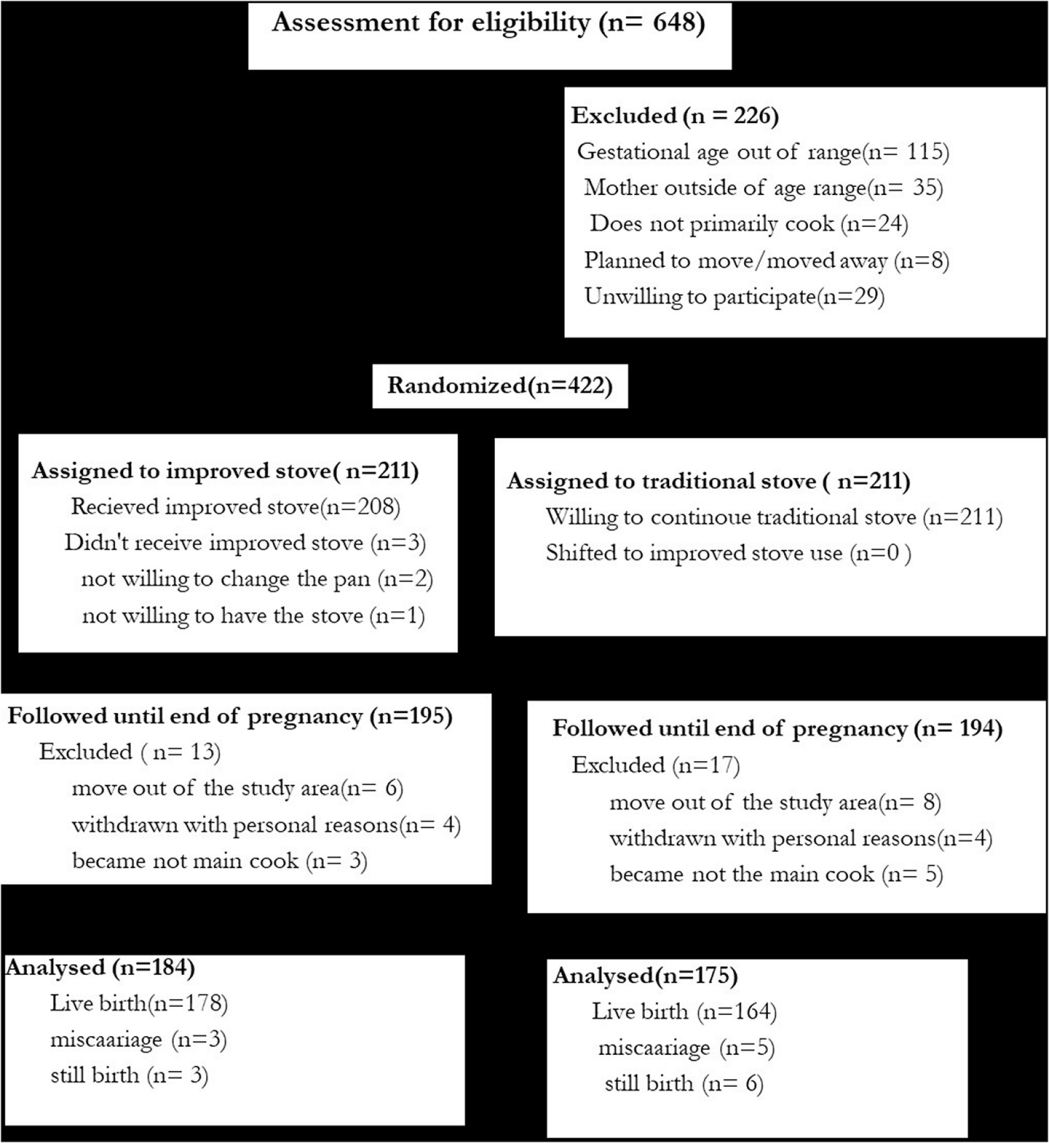
The Consolidated Standards of Reporting Trials (CONSORT) flow diagram

The author group has been updated above and the original article [1] has been corrected.
